# The Potential of cfDNA as Biomarker: Opportunities and Challenges for Neurodegenerative Diseases

**DOI:** 10.1007/s12031-025-02317-8

**Published:** 2025-03-13

**Authors:** Şeyma Aydın, Selçuk Özdemir, Ahmet Adıgüzel

**Affiliations:** 1 Department of Genetics, Faculty of Veterinary Medicine, Atatürk University, Erzurum, Turkey; 2 Department of Molecular Biology and Genetics, Faculty of Science, Atatürk University, Erzurum, Turkey

**Keywords:** Circulating biomarkers, Cell-free DNA, CfDNA, Liquid biopsy, Neurodegenerative disorders

## Abstract

Neurodegenerative disorders, including Alzheimer’s disease (AD), Parkinson’s disease (PD), multiple sclerosis (MS), and amyotrophic lateral sclerosis (ALS), are characterized by the progressive and gradual degeneration of neurons. The prevalence and rates of these disorders rise significantly with age. As life spans continue to increase in many countries, the number of cases is expected to grow in the foreseeable future. Early and precise diagnosis, along with appropriate surveillance, continues to pose a challenge. The high heterogeneity of neurodegenerative diseases calls for more accurate and definitive biomarkers to improve clinical therapy. Cell-free DNA (cfDNA), including fragmented DNA released into bodily fluids via apoptosis, necrosis, or active secretion, has emerged as a promising non-invasive diagnostic tool for various disorders including neurodegenerative diseases. cfDNA can serve as an indicator of ongoing cellular damage and mortality, including neuronal loss, and may provide valuable insights into disease processes, progression, and therapeutic responses. This review will first cover the key aspects of cfDNA and then examine recent advances in its potential use as a biomarker for neurodegenerative disorders.

## Introduction

The term “biomarker” is defined by the FDA-NIH Biomarker Working Group as “a characteristic that is measured as an indicator of normal biological processes, pathogenic processes, or responses to an exposure or intervention” (2016). Biomarkers hold considerable value due to their diagnostic, prognostic, and therapeutic potential (Califf 2018). In recent decades, extraordinary efforts have been made across all areas of clinical practice to identify new and more reliable biomarkers that are detectable with simple and non-invasive methods (Dhama et al. 2019; Hansson 2021). Circulating entities such as proteins, exosomes, and nucleic acids (cfDNA, mRNA, and miRNA) have drawn significant interest as biomarkers due to their accessibility and cost-effectiveness (Wieczorek and Reszka 2018; Dao et al. 2023). This review will focus on a circulating biomarker: cell-free DNA (cfDNA).

cfDNA was first detected in human plasma in 1948 (Mandel 1948); however, its potential clinical utility remained underappreciated until researchers observed distinctive differences in cfDNA levels and characteristics between healthy individuals and those with certain diseases. In a study in 1966, elevated cfDNA levels in patients with systemic lupus erythematosus (SLE) attracted interest in the potential use of cfDNA as a biomarker for autoimmune diseases (Tan et al. 1966). In 1977, it was reported that cancer patients had significantly elevated cfDNA levels in their serum compared to healthy individuals (S. A. Leon et al. 1977). Additionally, in 1994, point mutations of the N-RAS gene were detected in cfDNA derived from patients with acute myelogenous leukemia and myelodysplastic syndrome (Vasioukhin et al. 1994). As research has progressed, cfDNA has gained attention as a promising biomarker for diagnosing and monitoring a wide range of diseases, including fetal genetic abnormalities (Everett and Chitty 2015), solid tumors (Jung et al. 2010), rheumatologic diseases (Duvvuri and Lood 2019), cardiovascular conditions (Tan et al. 2023), and certain kidney disorders (Celec et al. 2018).

Timely and accurate diagnosis of neurodegenerative diseases is a pressing need in clinical practice. Biomarkers can improve the diagnostic workup of many neurodegenerative diseases, especially during the early stages. Numerous studies investigating cfDNA from patients with AD, PD, ALS, and MS suggest that cfDNA may serve as a valuable biomarker for detecting these disorders, monitoring treatment responses, and understanding the pathological mechanisms of neurological diseases.

## Molecular Mechanisms Governing the Release of cfDNA into Circulation

**Fig. 1:**
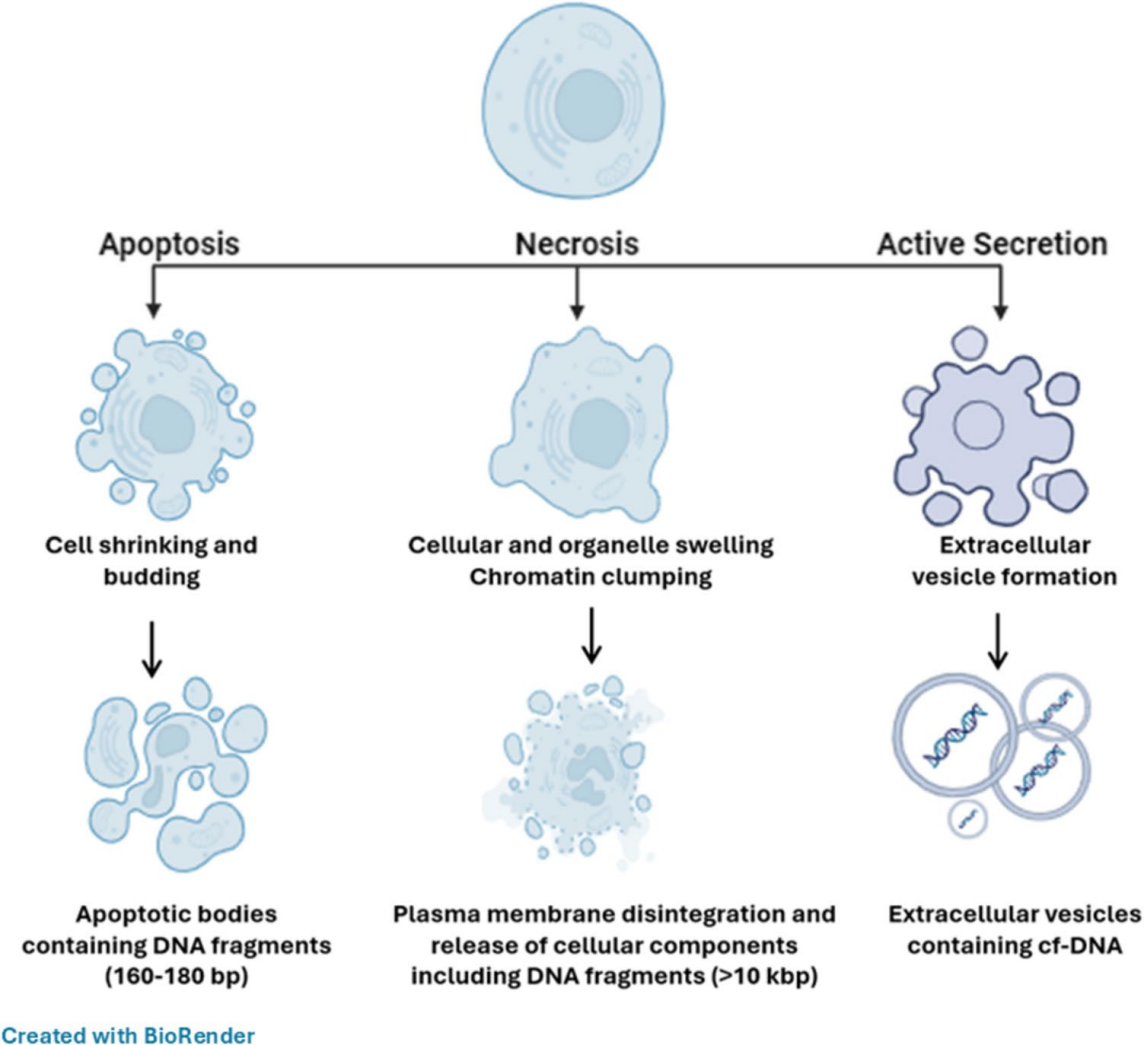
Cells release cfDNA through apoptosis, necrosis, and active secretion. In biofluids, cfDNA may exist as either free or associated with extracellular vesicles. There might be genetic and epigenetic alterations that can be found in cfDNA including mutations or methylations assisting detection of various disorders

Both normal and diseased cells release double-stranded short fragments, generally ranging from 80 to 200 bp, known as cfDNA. The presence of cfDNA has been detected in almost all bodily fluids such as plasma/serum, saliva, tears, urine, and cerebrospinal fluid (CSF) (Hui et al. 2015). In healthy individuals, cfDNA is present in bodily fluids at low concentrations. The concentration of cfDNA in the plasma of a healthy person is approximately 1 − 10 ng/mL, with the vast majority originating from the hematopoietic system (Yao et al. 2016). However, these levels can increase due to various physiological and pathological processes such as exercise (Hummel et al. 2018), aging (Teo et al. 2019), pregnancy (Bianchi and Chiu 2018), cancer (Mair and Mouliere 2021), tissue trauma (Brodbeck et al. 2019), inflammation (van der Meer et al. 2019), and sepsis (Charoensappakit et al. 2023). The releasing mechanism of cfDNA from cells to bodily fluids is poorly understood; however, apoptosis, necrosis, and active secretion are considered the major pathways of cfDNA release (Fig. 1) (Hu et al. 2021).

### Apoptosis

Apoptosis or programmed cell death is widely recognized as a major source of cfDNA release from healthy and diseased tissues (Ranucci 2019). Various stimuli and conditions, both physiological and pathological—such as hormonal regulation, DNA damage, and oxidative stress—can trigger this complex process, which involves a series of molecular events (Norbury and Zhivotovsky 2004). Caspases play a significant role during apoptosis by directing the morphological and biochemical changes such as membrane blebbing, cell shrinkage, chromatin condensation, and DNA fragmentation (Kiraz et al. 2016). Caspases activate a double-strand-specific endonuclease known as caspase-activated DNase (CAD), which contributes to cfDNA formation by cleaving chromosomal DNA into 160–180 bp nucleosomal fragments (Nagata 2000; Kustanovich et al. 2019). As a result of regular cleavage of DNA at internucleosomal regions during the apoptotic process, cfDNA derived from apoptosis typically exhibits a ladder-like pattern visible via gel electrophoresis (Wyllie et al. 1984).

DNA fragmentation may also occur in a caspase-independent manner if a cell cannot degrade its DNA due to a lack of functioning cell-autonomous caspases. In this situation, phagocytes and their waste-management nucleases will conduct engulfment-mediated DNA degradation (Heitzer et al. 2020). Therefore, DNA fragmentation during apoptosis can result both from CAD activity and from lysosomal acid DNase(s), most likely DNase II, after the apoptotic cells are engulfed (McIlroy et al. 2000).

Apoptotic cells are short-lived due to efficient clearance mechanisms, which exert strong anti-inflammatory and immunosuppressive effects (Savill and Fadok 2000). Impairments in apoptotic cell clearance and increased cell death are known to be associated with various human pathologies where elevated cfDNA levels can be detectable (Viorritto et al. 2007; Saukkonen et al. 2008; Hochreiter-Hufford and Ravichandran 2013).

### Necrosis

The detection of high-molecular-weight cfDNA in the body fluids of cancer patients suggests that necrosis could also be a mechanism for cfDNA release (Jahr et al. 2001). Necrosis is an accidental form of cell death caused by severe external damage to the cell (Syntichaki and Tavernarakis 2002). During necrosis, cells undergo several stages, including nuclear chromatin clumping, swelling of organelles and the cell, disintegration of the plasma membrane, and the eventual release of cellular components (Duvvuri and Lood 2019). In this process, chromatin is digested non-specifically, resulting in DNA fragments that are often around 10,000 base pairs in length, with some even larger (Jahr et al. 2001). At the end of necrosis, the membrane structure of dying cells is no longer intact, leading to the release of many intracellular substances, including high-molecular-weight DNA fragments, into the interstitial space (Galluzzi et al. 2014).

Necrotic release of cfDNA is commonly associated with conditions such as trauma, injury, and sepsis, with cfDNA levels correlating with the severity of these conditions (Charoensappakit et al. 2023). Despite necrosis being more common than apoptosis, the clearance of necrotic cells is slower, leading to a longer presence of these larger DNA fragments in the bloodstream (Viorritto et al. 2007). This cellular debris is difficult for phagocytes to clear, causing inflammation in the surrounding tissues (Gobeil et al. 2001).

### Active Secretion

Active secretion of cfDNA is a regulated process that can occur under various conditions and is believed to play a role in cell-to-cell communication, signaling, and other cellular functions (Bronkhorst et al. 2022). Insights into the active secretion of cfDNA from living cells come from experimental studies on cultured cells (Jahr et al. 2001; Stroun et al. 2001). Wang et al. (2017) determined that the release of cfDNA from cultured cells did not correlate with the level of cellular apoptosis or necrosis; instead, it was related to the percentage of cells in the G1 phase. They suggested that the increased release of cfDNA from differentiated cells might be due to its active secretion within exosomes, a type of extracellular vesicles (EVs), or other protective forms that prevent degradation in the blood.

EVs are spherical phospholipid-bilayered vesicles secreted by most cells known to play important roles in various physiological and pathological processes by mediating intercellular communication through transferring their biologically active components, including proteins, RNA, and DNA (Bazzan et al. 2021). cfDNA can be associated with various types of EVs including exosomes, apoptotic bodies, and microvesicles (Elzanowska et al. 2021; Kahlert et al. 2014). Fernando et al. (2017) extracted cfDNA from whole human blood plasma and plasma exosomes, compared their quantities, and showed that more than 90% of cfDNA is associated with exosomes.

Utilizing EVs for cfDNA extraction offers the advantage of protecting the DNA from degradation, thus ensuring a higher yield and better-quality samples. This makes EVs an attractive option for researchers and clinicians aiming to study cfDNA for diagnostic, prognostic, or therapeutic purposes in various disorders.

## Clearance Mechanisms of cfDNA

Impaired clearance mechanisms could be another factor causing elevated levels of circulating cfDNA (Nguyen et al. 2024). In healthy individuals, cfDNA is typically present at low levels due to the rapid clearance mechanism in which nucleases play a significant role (Han and Lo 2021). Research has shown that under physiological conditions, endonucleases swiftly degrade cfDNA, and subsequently, various organ systems, including the spleen, liver, and kidneys, take a role in elimination (Leung et al. 2016). Several factors, such as whether the cfDNA is bound to proteins, nucleosomes, or antibodies, or whether it is circulating freely or enclosed within membrane-bound particles like apoptotic bodies and exosomes, can affect the efficiency of endonucleases in clearing cfDNA (Leung et al. 2016). Rapid clearance is essential to maintain a balance between cfDNA release and removal, which helps prevent inflammation and the potential development of autoimmunity against DNA, as observed in SLE (Han and Lo 2021).

## Liquid Biopsy and the Emerging Significance of cfDNA

Tissue biopsy is considered the gold standard for biomarker testing due to the highly standardized methodologies used in molecular profiling. However, tissue-based assays have some limitations in the clinical setting such as insufficient DNA extraction from tissue samples due to inadequate tissue quantities (Ilié and Hofman 2016). Even when tissue is collected, DNA may become degraded or damaged due to preservation methods, potentially leading to false positive results in molecular assays (Wong et al. 2014). Additionally, tissue sampling may be unfeasible at diagnosis or during disease monitoring for various reasons, such as the risk of complications, tissue inaccessibility, or patient refusal (Schwarzenbach et al. 2011; Crowley et al. 2013).

In recent decades, accessible biofluids have attracted significant interest in determining and monitoring various disorders (Loy et al. 2024). Moreover, technological advances that have improved signal detection have enabled more informative and effective analysis of biological components in the biofluids (Gaitsch et al. 2023). Additionally, progress in the field of epigenetic profiling has expanded the use of liquid biopsies to a range of diseases that may lack distinct mutational profiles, such as autoimmune, inflammatory, infectious, and degenerative disorders. Thanks to these developments, a notable number of pathological conditions now benefit from enhanced diagnosis, prognosis, and treatment through the “liquid biopsy” approach.

The liquid biopsy technique involves sampling body fluids such as plasma, serum, CSF, urine, saliva, and pleural effusions to analyze molecular components released from cells, including free nucleic acids—such as cfDNA, cell-free RNA (cfRNA), and circulating tumor DNA (ctDNA)—and vesicles like circulating tumor cells (CTCs) and EVs (Bardelli and Pantel 2017; Di Sario et al. 2023). The liquid biopsy technique is a candidate to replace traditional tissue biopsies, offering several clinical advantages particularly when tissue sampling is difficult or nearly impossible, such as in the brain and spinal cord (Rapisuwon et al. 2016). As a non-invasive, cost-effective, safe, and easy-to-repeat method, liquid biopsy also offers a more comprehensive view of disease origins by analyzing genetic and epigenetic markers (Ghagane and Nerli 2024). Moreover, the readily available nature of some body fluids makes this approach even more beneficial, allowing for serial biopsies to monitor treatment effects over time (Goldberg et al. 2018). Various fields of medicine, including oncology, transplantation, and reproductive medicine, currently benefit from the liquid biopsy approach (Palmirotta et al. 2018; Verhoeven et al. 2018; Schobers et al. 2021).

Differential diagnosis of brain disorders can be difficult when imaging is not informative enough, and surgical biopsy is either impossible or not an acceptable option for the patient. As a result, a wide range of techniques, such as miRNA profiling, RNA sequencing, and methylation pattern analysis of samples acquired from liquid biopsy, have been used to detect neurological diseases (Malhotra et al. 2023).

cfDNA holds significant potential for detecting various disorders due to its ability to reflect genetic and epigenetic alterations. Although liquid biopsy is advantageous due to its safety and ease of collection, cfDNA levels, particularly the ones derived from the central nervous system (CNS), are typically very low in plasma, likely due to the blood–brain barrier (BBB) (McEwen et al. 2020). In this context, the major challenge in liquid biopsy lies not in sampling but in sample analysis (Cheung et al. 2018). Consequently, distinct measurement and detection methods are required to promote the use of cfDNA in clinical applications (Natalia et al. 2023).

## Approaches for Isolating and Detecting cfDNA

### Sample Collection

Circulating nucleic acids, including cfDNA, are highly sensitive to pre-analytical variability, which can affect their yield, integrity, genomic DNA contamination levels, and overall reliability (Song et al. 2022). Accurate detection and analysis of cfDNA depend heavily on proper sample collection and preservation techniques to ensure reliable molecular profiling. Key factors include the type of collection tube used, the duration and conditions of pre-centrifugation delays, handling and storage temperatures, centrifugation protocols and speeds, plasma storage duration, and the number of freeze–thaw cycles.

The selection of blood collection and storage tubes for cfDNA isolation should be based on the storage or transportation duration and the specific requirements of the analysis. Anticoagulant-coated blood collection tubes, such as EDTA, heparin, or citrate, are commonly used for collecting biofluids for cfDNA isolation, with EDTA being the most widely preferred option. For samples that can be processed within 4 to 6 h after collection and stored at room temperature, EDTA tubes are sufficient (Diaz et al. 2023). However, when blood processing is delayed (e.g., ≥ 6 h), specialized blood collection tubes, such as Streck Cell-Free DNA BCT and PAXgene Blood cfDNA Tubes, are recommended. These specialized tubes contain cell-stabilizing reagents that preserve cfDNA, minimize contamination from genomic DNA, and prevent leukocyte lysis during the storage and transport of whole blood (Zhang et al. 2022). They are particularly useful for maintaining cfDNA integrity for several days at room temperature (Sorber et al. 2020; Diaz et al. 2023), making them ideal for extended storage or transportation periods. While specialized tubes offer superior stability and reduced risk of genomic DNA contamination, they are at least 30 × more costly than EDTA tubes (Gerber et al. 2020). Therefore, their use should be prioritized in scenarios where delayed processing or extended transport times are anticipated.

The collection tubes mentioned above can also be used for CSF collection (Chai et al. 2024). CSF provides a more concentrated source of neuron-derived nucleic acids, offering greater sensitivity for disease-specific cfDNA analysis (Pingle et al. 2023). However, it is a relatively invasive method compared to blood collection. Obtaining CSF typically requires a lumbar puncture, an uncomfortable and invasive procedure that involves extracting fluid from the space surrounding the spinal cord (Hickman et al. 2023). Most published cfDNA studies on CSF have focused on neuro-oncology, as CSF is often collected during diagnostic lumbar punctures, providing an opportunity to analyze cfDNA content. In these cases, cfDNA is primarily used for mutation detection, with tumors being identified based on previously defined patient- or tumor-specific mutations (Iser et al. 2024; Otsuji et al. 2024; Cornelli et al. 2024).

A two-step centrifugation process involving an initial low-speed centrifugation at 800–2000 × g, followed by a high-speed centrifugation at 14,000–16,000 × g is widely recommended to ensure high-quality plasma preparation for cfDNA isolation, maximizing yield while minimizing contamination (Kerachian et al. 2021). The two-step approach effectively removes cellular debris and minimizes contamination from genomic DNA, preparing the sample for optimal cfDNA isolation.

If cfDNA isolation cannot be performed immediately, appropriate storage conditions are critical to preserving sample integrity. Short-term storage at − 20 °C (up to 48 h) or long-term storage at − 80 °C is recommended to prevent degradation (Peng et al. 2024). Studies have highlighted DNA losses during prolonged sample storage, emphasizing the importance of proper temperature management (Gerber et al. 2020; Danesi et al. 2021). Additionally, repeated freeze–thaw cycles should be avoided to prevent further fragmentation of cfDNA, which could compromise downstream analysis (Greytak et al. 2020).

### Isolation Methods for cfDNA

For precision medicine, efficient isolation techniques for cfDNA extraction are essential, as cfDNA is present at very low concentrations in biofluids and can be easily contaminated with other molecules. The choice of pre-analytical protocols and workflows, including cfDNA isolation, can significantly impact fragmentation, yield, and quality, which, in turn, influence the conclusions drawn from biomarker testing (Gong et al. 2022; van der Pol et al. 2022).

Several methods can be used to extract cfDNA, including columns, magnetic beads, phenol–chloroform-based methods, and commercial spin column kits. Selecting an appropriate cfDNA isolation method depends on the intended application, sample type, and available resources. For routine clinical diagnostics, methods such as magnetic beads and spin column kits are preferred due to their reliability and compatibility with automated systems.

The magnetic bead technique is highly efficient in capturing fragmented DNA, even from low-concentration samples, and can provide consistent performance across a range of biofluids. However, this method is moderately expensive due to the cost of beads and associated reagents. Spin column kits with silica membranes are widely used in research studies due to their simplicity, high yield, and ability to remove contaminants. These kits are reliable for both research and diagnostic purposes. While they have a higher cost per sample compared to other methods (Zamuner et al. 2024), their ease of use and robustness make them a justified expense in clinical settings. Although spin column-based methods are typically more costly and time-consuming than magnetic approaches, they typically produce higher yields (Polatoglou et al. 2022).

The phenol–chloroform-based method is effective for extracting cfDNA (Khurram et al. 2023), but the process is labor-intensive and prone to contamination. Additionally, organic solvents can interfere with downstream applications. While the method uses low-cost reagents, its manual nature increases labor costs and reduces throughput, limiting its clinical utility. To address the problems caused by organic solvents, Das et al. developed a phenol–chloroform-free method, which provides a cost-effective, safe, and rapid cfDNA extraction solution optimized for cell-conditioned media. However, this method has not yet been optimized for plasma and/or serum samples. In general, the developed method can be used to isolate cfDNA from various in vitro cell culture models (Das et al. 2024).

Additionally, emerging approaches, such as the microfluidic technique (Xu et al. 2019) and liquid-phase-based DNA isolation methods (Janku et al. 2021), show promise for achieving high sensitivity and specificity in cfDNA isolation. However, the application of these methods in cfDNA isolation and analysis still requires further exploration. Currently, these methods are in the experimental stages, but they offer the potential for automation and minimal sample loss.

### Detection Methods for cfDNA

Various methods can be used including quantitative PCR (qPCR), digital PCR (dPCR), and next-generation sequencing (NGS) technologies to accomplish sensitive and specific detection of cfDNA. Since each method has its own advantages and limitations, selecting the appropriate method depends on the specific application and the required sensitivity.

The sensitivity of cfDNA detection varied across different techniques, with NGS demonstrating the highest sensitivity, ddPCR, and then qPCR (Naegele et al. 2024). While the sensitivity of NGS was the highest, the specificity of all three methods (NGS, ddPCR, and qPCR) was found to be similar (Naegele et al. 2024). These findings highlight the enhanced sensitivity of ddPCR relative to qPCR, and the superior sensitivity of NGS, suggesting that NGS may be the most effective tool for sensitive detection, while ddPCR remains a highly sensitive alternative to qPCR.

The qPCR method utilizes fluorescent markers that bind to DNA, with fluorescence intensity directly correlating to the present DNA quantity. As a cornerstone technique for cfDNA detection, qPCR enables real-time quantification of specific DNA sequences. The qPCR method is user-friendly and cost-effective and offers a quick turnaround time. Its accessibility and compatibility with standard qPCR equipment in most laboratories make it a valuable tool for detecting known mutant genes (Bohers et al. 2021). Additionally, it can be adapted to analyze epigenetic patterns of cfDNA using methylation-specific primer designs targeting loci identified in array and sequencing studies (Šestáková et al. 2019). Widely accessible and cost-effective, qPCR remains a popular choice for cfDNA detection. However, its ability to detect rare mutations or multiple targets simultaneously is limited. Recent studies suggest that dPCR and ddPCR offer greater measurement reliability than qPCR especially when analyzing samples with low cfDNA concentrations (Moreno-Manuel et al. 2021; Clausen et al. 2023).

dPCR was designed to enhance analytical sensitivity and precision by dividing the input sample into a large number of individual reactions prior to amplification, enabling absolute quantification (Moreno-Manuel et al. 2021). This unique capability makes it particularly valuable for liquid biopsy applications. Unlike qPCR, dPCR provides absolute quantitative results without relying on standard curves and demonstrates superior precision, reproducibility, and sensitivity (Wainman et al. 2024). These attributes make it ideal for analyzing small nucleic acid inputs, as well as detecting low-abundance mutations and copy number variations (CNVs) in cfDNA with exceptional accuracy.

However, dPCR can only detect known mutations, precluding the identification of novel genetic alterations. Additionally, its multiplexing capacity is relatively low compared to NGS. While ongoing advancements in multiplex dPCR assays show promise, current applications are still limited in this regard. Moreover, the technique requires specialized equipment, trained personnel, and standardized protocols, which can hinder its accessibility and consistency in clinical and research settings. However, dPCR remains a powerful tool for monitoring patients and assessing treatment responses over time, owing to its highly sensitive quantification of mutated alleles at a relatively low cost (Fernández-Lázaro et al. 2020). It is particularly valuable for applications where detecting and quantifying minor mutations is critical, such as in liquid biopsies for oncology or precision medicine.

NGS technologies have revolutionized genome analysis, enabling broad-scale and in-depth genetic studies that were previously unattainable. NGS offers a highly sensitive method capable of detecting a wide array of genetic alterations, providing a comprehensive genetic landscape. By sequencing millions of DNA fragments in parallel, NGS facilitates detailed analysis of cfDNA, making it particularly useful for monitoring therapy response and identifying resistance mechanisms early in treatment. This method can be applied to targeted panels for highly specific and sensitive detection of cfDNA mutations while also enabling the identification of multiple mutations and epigenetic changes, such as DNA methylation patterns (Birknerova et al. 2022). The advanced multiplexing capabilities of NGS allow for the simultaneous analysis of hundreds of genetic targets, offering broad coverage with improved sensitivity. Modern NGS systems, such as those developed by Illumina, boast high accuracy and reduced marginal costs per read, making them widely used for cfDNA analysis (Song et al. 2022). Furthermore, “methylation profiling” via NGS is gaining attraction, enabling researchers to identify epigenetic changes linked to disease onset and progression.

High costs, long turnaround times, and significant computational and bioinformatic requirements present challenges for routine clinical use of NGS. Extensive quality control measures and technical expertise are also necessary for reliable data interpretation. However, recent research shows that targeted panel testing reduces costs compared with conventional single-gene biomarker (Naegele et al. 2024). Moreover, when holistic testing costs (eg, turnaround time, technical expertise costs, number of hospital visits) are considered in the analysis, targeted panel testing consistently provides cost savings versus single-gene testing.

As NGS costs continue to decline, its utility in clinical settings is expected to expand. In comparison, dPCR offers a targeted approach to analyze a small number of known and selected biomarkers. While NGS provides broad-ranging information and is ideal for diagnostic purposes, dPCR’s precision and affordability make it a powerful tool for specific applications such as monitoring known mutations or selected biomarkers. The complementary use of dPCR and NGS is emerging as a promising strategy, with dPCR offering rapid, sensitive validation and NGS providing comprehensive data for diagnosis and biomarker discovery.

## Emerging Role of cfDNA as a Biomarker for Neurodegenerative Diseases

Many brain disorders show slow progression with a long asymptomatic stage followed by a mild clinical symptom period. Due to the nonspecific clinical symptoms and the lack of reliable biomarkers, timely and accurate diagnosis of many brain disorders presents a challenge (Voigtlaender et al. 2024). Traditional diagnostic methods used in clinical practice are often invasive and time-consuming, with limited specificity and sensitivity for early-stage detection (Malhotra et al. 2023). The late detection of diseases limits treatment efficiency, highlighting the need for the development of noninvasive and reliable diagnostic tools. Early diagnosis of neurological disorders is essential for improving patient care and evaluating treatment responses, addressing a critical unmet medical need (Hansson 2021). Due to the lack of diagnostic biomarkers for many disorders—except in rare cases where causative genetic mutations are detectable—the identification of specific biomarkers, including biofluid and molecular imaging markers, remains a major research priority (TCW and Goate 2017; Gijselinck et al. 2018; Ghasemi and Brown 2018). Several studies have explored the role of cfDNA in diagnosing and understanding neurodegenerative diseases with a primary focus on concentration quantification and methylation analysis (Loft et al. 2023).

Imaging techniques such as MRI, PET, and CT provide valuable insights into structural, metabolic, and functional brain changes, with high sensitivity for advanced neurodegenerative conditions, such as brain atrophy in AD and substantia nigra degeneration in PD (Abhisheka et al. 2024). However, these techniques have limited sensitivity for detecting early-stage or subtle changes. PET scans with disease-specific tracers (e.g., amyloid or tau in AD) offer high specificity, while MRI and CT have moderate specificity, as structural changes may overlap with conditions like vascular dementia or aging (Lee et al. 2024). Although imaging allows for disease staging and monitoring progression, it is time-consuming, requires expensive equipment, and sometimes involves the use of radioactive tracers (Xie et al. 2024). In contrast, cfDNA assays, once validated, could provide a non-invasive, cost-effective, and scalable alternative for early detection and treatment monitoring. cfDNA can be detected months before radiological changes and is useful for multiple non-invasive, longitudinal assessments (Li et al. 2024b). However, cfDNA lacks anatomical or spatial information, which limits its use in disease staging. Combining imaging techniques for spatial resolution with cfDNA for early detection and screening could optimize the diagnosis and management of neurodegenerative diseases.

The short half-life of cfDNA (approximately 2 h) enables it to reflect dynamic changes in disease-related pathological conditions accurately (Loft et al. 2023). It can also fluctuate in response to therapeutic interventions (Cisneros-Villanueva et al. 2022) and serve as a real-time biomarker that reflects ongoing changes in disease activity and neuronal damage. This is especially useful for monitoring therapeutic efficacy in diseases like AD or PD.

cfRNA (including microRNAs (miRNAs), long non-coding RNAs (lncRNAs), and messenger RNAs (mRNAs)) is attracted attention as a valuable biomarker in neurodegenerative disorder research, as it reflects dynamic gene expression changes associated with disease progression, offering a real-time snapshot of biological processes (Zhong et al. 2024). However, cfRNA is more prone to rapid degradation compared to cfDNA, making cfDNA easier to analyze and transport. Due to its instability, cfRNA requires careful handling and highly sensitive techniques, while cfDNA is more robust and suitable for routine analysis (Ma et al. 2024). Additionally, cfDNA provides insights into both epigenetic and genetic alterations, such as genetic mutations and methylation patterns.

Abnormalities in DNA methylation patterns are associated with several diseases including neurological disorders (Zemmour et al. 2018; Siejka-Zielińska et al. 2021; Bahado-Singh et al. 2022). Therefore, cfDNA can be analyzed for methylation status to determine abnormal patterns that aid in the diagnosis of neurological diseases. cfDNA methylation markers for tau pathology in AD are suggested to provide higher specificity compared to some protein biomarkers that overlap between neurodegenerative conditions (Khemka et al. 2024). Additionally, methylation patterns of cfDNA can be used to reveal its tissue and cellular source thereby facilitating the diagnosis of neuron-specific damage (Bahado-Singh et al. 2022). In a study, Pollard et al. (2023) utilized neuron-derived cfDNA to accurately identify patients with AD by detecting neuron-derived cfDNA by identifying differentially methylated regions between neurons and blood plasma and have provided evidence of the feasibility of this approach to detect and monitor the onset and progress of neurological disorders.

Additionally, the fragmentation pattern of cfDNA may allow for the distinction of cfDNA from different origins (Ding and Lo 2022). Studies have reported that circulating tumor-derived cfDNA molecules have a shorter size distribution compared to the background cfDNA, which is primarily of hematopoietic origin, suggesting that a fragment size-based approach could enhance the accuracy of data interpretation (Mouliere et al. 2018).

Mitochondrial dysfunction can accelerate neuronal degeneration and contribute to disease progression because mitochondria are involved in cellular energy production, calcium homeostasis, and cell survival pathways (Klemmensen et al. 2024). The brain is one of the most energy-demanding organs in the human body, and many neuropsychiatric and non-psychiatric neurological diseases have mitochondrial dysfunction associated with disease pathophysiology (Choi et al. 2024). The reciprocal relationship between mitochondrial dysfunction and mitochondrial genome integrity in several neurodegenerative diseases has been extensively characterized in the last decades (Peggion et al. 2024). This has prompted the need to clarify the role of mtDNA as an indicator of the pathogenic mechanisms underlying neurodegeneration. Circulating cell-free mitochondrial DNA (ccf-mtDNA) originating from dysfunctional mitochondria can be detected in biofluids (Risi et al. 2025). Research determined that cf-mtDNA can provoke mechanisms including immune and inflammatory responses seen in neurodegenerative disorders such as PD and AD (Podlesniy et al. 2013; Lowes et al. 2020; Borsche et al. 2020). In the case of acute brain injury, elevated levels of cf-mtDNA correlated with clinical severity and interleukin-6 (IL-6) cytokine response were found in CSF and serum samples (Kayhanian et al. 2019). In another study, a positive correlation was determined between CSF cf-mtDNA and various comorbidities such as depression and insomnia; however, these levels were only significant if measured in the absence of treatment (Lowes et al. 2019).

Currently, FDA-approved cfDNA blood-based tests are available for colorectal cancer screening in eligible populations (Chung et al. 2024). Additionally, numerous clinical trials are ongoing to evaluate cfDNA-based cancer detection methods (Medina et al. 2023). Despite substantial research on cfDNA as a biomarker for neurodegenerative diseases, data from PubMed, clinicaltrials.gov, FDA approvals, and company newsletters showed there is no cfDNA-based biomarker has been approved for the diagnosis of AD, PD, ALS, or MS. This highlights a significant gap in translating cfDNA research into clinical applications for these disorders, underscoring the need for further validation and development.

### Alzheimer’s Disease (AD)

AD is a severe neurodegenerative disorder of the brain that is marked by the pathological aggregation of amyloid-beta plaques and tau tangles, which ultimately lead to neurodegeneration (Assfaw et al. 2024). Clinically, AD manifests as cognitive decline, dementia, alterations in higher-order brain functions, and, in some cases, movement disorders (Kovacs 2019). Most patients remain asymptomatic during the preclinical stage; therefore, early detection is critical for the treatment or use of disease-modifying drugs (Cummings 2021). However, early diagnosis poses significant challenges. Current diagnostic approaches primarily rely on determining mental decline, which often leads to late-stage diagnosis when extensive brain damage has already occurred due to progressive neuronal cell death (Perluigi et al. 2024). Consequently, therapeutic interventions aimed at preventing the disease are often ineffective once symptoms manifest (Fish et al. 2019). Existing treatments provide modest symptomatic relief, typically extending quality of life by only a few years before significant cognitive deterioration sets in Weller and Budson (2018). Early pre-symptomatic diagnosis has the potential to revolutionize AD management, enabling interventions that could halt disease progression and significantly improve patient outcomes. Recognizing this, considerable efforts have been directed toward developing biomarker-based approaches to facilitate earlier detection.

As a biomarker, circulating cfDNA can be one promising avenue in detecting and tracking AD. Significantly elevated levels of circulating cfDNA have been observed in AD patients compared to controls, suggesting heightened neuronal damage and underscoring the utility of cfDNA as a marker for neurological disorders (Nguyen et al. 2024).

DNA methylation changes relevant to AD have recently been identified in human brain tissue (Macías et al. 2023). This has prompted research to investigate whether such methylation changes can be detected in plasma cfDNA from AD patients and controls. If successful, this approach could provide access to epigenetic information otherwise “locked” within the brain, offering a non-invasive window into disease-specific processes.

A study investigating DNA methylation in CYP genes using circulating cfDNA from Alzheimer’s disease (AD) patients identified significant CpG site changes in CYP51A1 and CYP2S1. These genes play critical roles in cholesterol metabolism, neurotoxicity, and drug metabolism, processes closely associated with AD pathogenesis (Bahado-Singh et al. 2023).

Another study reported changes in the methylation patterns of *PRLHR*, a gene involved in energy balance regulation. Dysregulation of energy homeostasis is closely related to AD development, making PRLHR methylation changes in plasma cfDNA of living AD patients a promising marker for the disease (Macías et al. 2023).

Further research by Ding et al. explored mtDNA methylation alterations in plasma cfDNA. Their work highlighted distinct methylation patterns that differentiate AD patients from healthy controls, emphasizing mtDNA methylation’s diagnostic potential. Specifically, the study identified hypomethylation in the non-protein-coding regions of mtDNA, suggesting that this epigenetic alteration may contribute to AD pathogenesis. These findings indicate that mtDNA hypomethylation could serve as both a biomarker for AD and a target for further investigation into the molecular mechanisms underlying the disease (Ding et al. 2023).

The APOE4 allele is a well-established genetic risk factor for sporadic AD, substantially increasing an individual’s susceptibility to the disease (Norwitz et al. 2021). The presence of the APOE ε4 allele has been closely linked to both early-onset and progressive forms of AD, primarily through its influence on amyloid-beta (Aβ) aggregation, clearance, and lipid metabolism, as well as its role in neuroinflammation. In a recent study, Macías et al. (2024) investigated the potential of detecting APOE ε4 allele-specific DNA in cfDNA as a complementary approach to traditional AD biomarkers, such as Aβ and tau proteins. Their work highlights the possibility of using cfDNA as a non-invasive tool to assess genetic risk, particularly in individuals who may not yet exhibit clinical symptoms of AD. By analyzing cfDNA, the study provided evidence that APOE ε4 allele-specific DNA can be reliably detected and monitored, paving the way for its integration into liquid biopsy approaches for AD diagnostics.

Together, these studies underscore the utility of cfDNA analyses, particularly in nuclear and mitochondrial DNA, as a less invasive tool for AD diagnosis and disease monitoring. They also pave the way for a better understanding of the epigenetic changes that influence AD development and progression.

### Parkinson’s Disease (PD)

PD is linked to the accumulation of alpha-synuclein protein and the excessive loss of dopaminergic neurons in the substantia nigra (Trist et al. 2019). The loss of dopamine-producing neurons leads to symptoms such as muscular rigidity, resting tremors, bradykinesia, postural instability, and gait impairment (Reddy et al. 2024). As with many neurodegenerative diseases, the symptoms of PD appear well after the pathology has begun, due to the brain’s compensatory mechanisms (Johansson et al. 2024). This delayed onset of symptoms, combined with the extensive neuronal cell death that occurs in advanced stages, makes treatment challenging (Armstrong and Okun 2020). Accurate diagnosis remains difficult, and identifying the early stages of the disorder is a focus of ongoing research. A simple and non-invasive method for early diagnosis could significantly enhance the effectiveness of the limited treatment options currently available.

cfDNA is being examined as a biomarker for neuronal injury in PD. Limited reports have primarily focused on levels of cf-DNA and cf-mtDNA in CSF and serum of PD patients. Quantitative analysis of serum cf-DNA in patients revealed significantly elevated levels of both cf-mtDNA and total cf-DNA compared to healthy controls (Wojtkowska et al. 2024). Analyzing serum circulating cell-free nuclear DNA levels by measuring the expression of the nuclear gene *KRAS*, Wojtkowska et al., revealed a slight but significant increase in PD patients. However, the increase of cf-nDNA level in PD patients disappeared after adjusting for age (Wojtkowska et al. 2024).

Elevated cf-mtDNA levels in PD patients point to mitochondrial dysfunction and oxidative stress as contributing factors in the disease. Elevated levels of serum cf-mtDNA and IL-6 were detected in patients with biallelic *PINK1* and *Parkin* mutations (Borsche et al. 2020). This outcome shows there is an association between increased cf-mtDNA release and heightened neuroinflammation in these cases. On the other hand, a study reported CSF cf-mtDNA levels are significantly reduced in PD patients compared to matched controls (Lowes et al. 2020). These findings suggest potential compartment-specific differences in cfDNA dynamics within the body.

Mitochondrial dysfunction is present in both idiopathic Parkinson’s disease (iPD) and LRRK2-related Parkinson’s disease (LRRK2-PD), but distinct mitochondrial pathologies may underlie these disorders. Using a novel multiplex digital PCR assay to quantify cf-mtDNA copy numbers and deletion ratios in cerebrospinal fluid (CSF), researchers found significant differences between iPD and LRRK2-PD patients. Notably, high cf-mtDNA deletion levels were observed in iPD but not LRRK2-PD, with these findings validated in an independent cohort. This suggests that cf-mtDNA content and deletion ratios may help differentiate between iPD and LRRK2-PD and highlight differing mitochondrial pathophysiology in these diseases (Puigròs et al. 2022).

Differential DNA methylation in cfDNA has also been studied as a diagnostic tool. Whole-genome bisulfite sequencing (WGBS) of cfDNA in CSF revealed distinct methylation profiles in PD patients (Meng et al. 2021).

### Amyotrophic Lateral Sclerosis (ALS)

ALS is marked by the loss of motor neurons, resulting in increasing muscular weakening, progressive paralysis, muscle atrophy, and ultimately death due to respiratory failure (El Khalfi et al. 2024). The mean survival time after symptom onset is approximately 36 months, and due to the lack of specific biomarkers for ALS, the average diagnostic delay is about 12 months (Masrori and Van Damme 2020). ALS can present with behavioral and cognitive abnormalities, further complicating diagnosis and treatment (Srinivasan et al. 2024). Identifying specific biomarkers remains a critical challenge in ALS research, as early detection could assist in early diagnosis and increase access to clinical trials.

cfDNA presents a promising non-invasive approach for evaluating neuronal mortality in ALS patients. Mitochondrial impairment is a key factor in ALS pathogenesis, as cf-mtDNA is particularly susceptible to oxidative stress due to the lack of histone protection and an inefficient repair system (Newman and Shadel 2023). Patients with ALS demonstrate elevated amounts of circulating cf-mtDNA. This heightened vulnerability may lead to the release of cf-mtDNA fragments into the circulation (Gambardella et al. 2019).

Recent research highlights that misfolded proteins like SOD1 and TDP-43 can disrupt mitochondria, triggering cytoplasmic release of mtDNA and activating immune responses in ALS (Tan et al. 2022). In a study, it was found elevated serum cf-mtDNA in ALS patients, suggesting its link to mitochondrial dysfunction and inflammatory processes (Li et al. 2024a). While cf-mtDNA levels were not significantly associated with ALS severity, they correlated with disease progression, particularly in patients with SOD1 mutations. Additionally, mutations in ALS-associated genes (C9orf72, SOD1, and TARDBP) are detectable in cfDNA and may aid in distinguishing familial and sporadic ALS forms (Li et al. 2024a).

There is growing clinical evidence for the potential of DNA methylation, especially cfDNA methylation, to enhance the diagnosis of ALS (Robichaud et al. 2021). Several methods, based on genome-wide DNA methylation profiling, can be performed to uncover complex new specific signatures that may improve the diagnosis of ALS. Research has revealed altered methylation signatures in ALS patients compared to healthy individuals (Coppedè 2024). Overall, the progress presented in this work that highlights the clinical potential of DNA methylation and circulating cfDNA as diagnostic biomarkers in various conditions should serve as further motivation for their study in ALS (Robichaud et al. 2021).

ALS lacks specific biomarkers, but the selective toxicity to motor neurons (MNs) provides an opportunity to detect MN-derived cfDNA in biofluids. Using tissue-informative markers (TIMs) identified through WGBS, cfDNA from ALS patients and controls was bisulfite-treated, hybridized to capture probes, and sequenced. Computational analysis and machine learning identified methylation features associated with ALS. This study highlights a novel cfDNA methylation capture panel as a promising non-invasive diagnostic tool for ALS, with the potential to enable early intervention and personalized treatment. Further validation is required to refine its clinical application (Caggiano et al. 2024).

### Multiple Sclerosis (MS)

MS is a chronic, autoimmune, and neurodegenerative disease that arises as a result of the immune system mistakenly attacking the protective covering of nerve fibers and results in disrupting communication between the brain and other body parts (Liang et al. 2024). The etiological ambiguity, coupled with the clinical variability of the disease, creates substantial diagnostic and prognostic challenges, delaying early diagnosis and treatment (Ford 2020). Currently, there are no accurate predictors of neurodegeneration in MS (Harris et al. 2017). The diagnostic process remains lengthy and costly, underscoring the urgent need for highly sensitive and specific diagnostic and prognostic tests that are preferably minimally invasive.

A study found significantly higher concentrations of cfDNA in MS patients compared to healthy individuals. Additionally, similar to other neurological disorders, analyzing the methylation status of circulating cfDNA could facilitate the development of molecular markers for MS (Dunaeva et al. 2018).

Mitochondrial dysfunction is closely linked to multiple sclerosis (MS), and altered levels of cell-free mitochondrial DNA (cf-mtDNA) may serve as indicators of neurodegeneration in MS (Lowes et al. 2019). This study explored the roles of circulating HMGB1, mtDNA, and pro-inflammatory cytokines in neuroinflammation associated with progressive MS. Results showed that plasma mtDNA levels were higher in secondary progressive (SP) MS compared to primary progressive (PP) MS, whereas HMGB1 levels did not differ significantly between the two forms. Pro-inflammatory cytokines, including TNF-α, IL-1β, and IL-6, were elevated in progressive MS patients, with TNF-α correlating with disease severity in PP MS. These findings indicate that cf-mtDNA and cytokines contribute to systemic inflammation in progressive MS, with distinct activation patterns in SP and PP forms. Further investigation is required to elucidate their roles and potential as therapeutic targets (Nasi et al. 2020).

## Challenges and Priority Recommendations

Circulating cfDNA derived from neuronal or glial cells has emerged as a potential biomarker for disease-associated cellular damage, reflecting the pathophysiological processes of neurodegenerative diseases such as AD, PD, MS, and ALS. cfDNA is considered a less invasive method compared to traditional techniques like brain imaging or biopsies, offering a means to detect neurodegeneration through blood samples. However, the application of cfDNA in clinical practice faces several significant challenges.

The clinical application of cfDNA faces additional challenges, including its short half-life, contamination with genomic DNA, and the lack of standardized pre-analytical steps. These issues necessitate the development of international guidelines for cfDNA handling and analysis to ensure consistency and reproducibility. The National Cancer Institute (NCI) has established guidelines for the collection, processing, storage, and quality assessment of blood samples for cfDNA analysis in cancer research. Similar standards for neurodegenerative diseases are needed to provide a framework for researchers and clinicians, ensuring that cfDNA analysis in these contexts is conducted in a reliable and reproducible manner.

Another significant challenge is the low concentration of CNS-derived cfDNA in the bloodstream, which complicates detection. Neuronal cfDNA is often present in very small amounts, making it difficult to distinguish from cfDNA released by other cell types, particularly during systemic inflammation, cancer, or other diseases. This can lead to ambiguity in interpreting cfDNA levels in the context of neurodegenerative disorders. Moreover, cfDNA levels are subject to substantial variability among individuals due to factors such as age, gender, medication use, physical activity, and diet.

These factors not only influence the concentration of cfDNA but may also affect the methylation patterns or fragmentation profiles of the DNA, further complicating analysis. Current technologies such as ddPCR and NGS are improving the sensitivity and specificity of cfDNA detection, but these methods still require further optimization to meet the clinical requirements for precision diagnostics. Furthermore, the lack of standardized protocols for sample collection, processing, and analysis contributes to variability in results across different studies and laboratories. This lack of standardization is a major barrier to the widespread adoption of cfDNA-based diagnostics in clinical practice, as inconsistencies in methodology can lead to unreliable or non-reproducible findings.

A critical issue in enhancing the utility of cfDNA as a diagnostic tool is differentiating neuron-derived cfDNA from cfDNA originating from other tissues. Such differentiation could significantly improve the specificity of cfDNA-based diagnostics by providing a clearer indication of neuron-specific damage. This approach would enable more precise identification of neurological conditions, allowing for earlier detection and better monitoring of disease progression. Currently, while the tissue of origin of cfDNA can often be inferred through certain markers, more refined methods are required to enable robust, neuron-specific detection.

Another promising direction to enhance diagnostic precision is the concept of multimodal or multi-analyte liquid biopsy approach which integrates cfDNA with other biomarkers, such as proteins (e.g., tau, amyloid-beta) or imaging techniques (e.g., PET scans). Combining cfDNA with protein biomarkers could provide deeper insights into disease mechanisms and improve the accuracy of diagnoses. For example, amyloid-beta and tau proteins are key biomarkers for Alzheimer’s disease, and pairing their analysis with cfDNA could help pinpoint early neurodegenerative changes. Moreover, imaging modalities like PET scans can confirm and localize structural or metabolic changes in the brain, complementing cfDNA analysis to improve disease staging and therapeutic guidance.

The integration of artificial intelligence (AI) and machine learning (ML) into the analysis of cfDNA and other biomarkers holds transformative potential. AI and ML techniques can handle large, complex datasets generated from omics experiments, such as transcriptomics, epigenomics, and proteomics, and integrate them with clinical and imaging data. By leveraging AI, researchers can uncover meaningful correlations and causal relationships between biomarkers and disease states, improving the precision of early diagnosis, disease subtyping, and prediction of disease progression. AI-based clinical decision support systems (CDSS) could also enhance the application of cfDNA and other biomarkers in clinical practice, facilitating the development of predictive models and personalized treatment strategies.

Reflecting mitochondrial dysfunction cf-mtDNA is particularly promising for neurodegenerative diseases as mitochondrial dysfunction is a hallmark of many neurodegenerative disorders. Measuring mtDNA could provide valuable insights into disease progression and therapeutic efficacy. Thus, more research is needed to validate the use of mtDNA as a biomarker in neurodegenerative diseases. The identification of mitochondrial subtypes within diseases could also pave the way for novel precision medicine strategies aimed at counteracting neurodegeneration.

Another main challenge is the insufficient validation of cfDNA markers across diverse populations and disease contexts. Additionally, integrating cfDNA-based diagnostics into clinical workflows involves logistical, economic, and regulatory challenges. Large-scale, multicenter studies are needed to validate cfDNA biomarkers in various demographic groups and clinical conditions, ensuring their robustness and clinical applicability. Furthermore, developing decision-support tools and training programs for healthcare providers will be essential for facilitating the adoption of cfDNA-based diagnostics in clinical settings. By addressing these challenges and fostering collaboration among researchers, clinicians, and regulatory agencies, the field of cfDNA research can progress toward realizing its full potential in transforming clinical practice, particularly in the diagnosis and management of neurodegenerative diseases.

## Conclusion

cfDNA-based biomarkers are emerging as promising tools for diagnosing and monitoring neurodegenerative disorders involving complex pathologies. Limited research on AD, PD, ALS, and MS has highlighted the potential of cfDNA, particularly in reflecting neuronal injury and mitochondrial dysfunction. Despite ongoing obstacles such as low concentrations and specificity, advancements in detection methods, such ddPCR and NGS, are addressing some of these constraints. Large multicenter trials with diverse participant populations are crucial to achieving more accurate and reproducible results trials are required to completely ascertain the clinical value of cfDNA biomarkers for neurodegenerative disorders. Additionally, extensive validation studies are required to authenticate cfDNA indicators in varied patient demographics and across multiple neurodegenerative disorders. Despite pre-analytical and technical challenges, liquid biopsy is emerging as a promising technique to further explore neurodegenerative diseases.

## Data Availability

No datasets were generated or analysed during the current study.

## Abbreviations

AD: Alzheimer’s disease
PD: Parkinson’s disease
MS: Multiple sclerosis
ALS: Amyotrophic lateral sclerosis
cfDNA: Cell-free DNA
ccf-mtDNA: Circulating cell-free mitochondrial DNA
CSF: Cerebrospinal fluid
SLE: Systemic lupus erythematosus
CAD: Caspase-activated DNase
EVs: Extracellular vesicles
CNS: Central nervous system
qPCR: Quantitative PCR
ddPCR: Droplet digital PCR
BEAMing: Beads, emulsions, amplification, and magnetics
NGS: Next-generation sequencing
